# Mitofusin 2 Deficiency Affects Energy Metabolism and Mitochondrial Biogenesis in MEF Cells

**DOI:** 10.1371/journal.pone.0134162

**Published:** 2015-07-31

**Authors:** Maria Kawalec, Anna Boratyńska-Jasińska, Małgorzata Beręsewicz, Dorota Dymkowska, Krzysztof Zabłocki, Barbara Zabłocka

**Affiliations:** 1 Molecular Biology Unit, Mossakowski Medical Research Centre, PAS, Warsaw, Poland; 2 Department of Biochemistry, Nencki Institute of Experimental Biology, PAS, Warsaw, Poland; University of Palermo, ITALY

## Abstract

Mitofusin 2 (Mfn2), mitochondrial outer membrane protein which is involved in rearrangement of these organelles, was first described in pathology of hypertension and diabetes, and more recently much attention is paid to its functions in Charcot-Marie-Tooth type 2A neuropathy (CMT2A). Here, cellular energy metabolism was investigated in mouse embryonic fibroblasts (MEF) differing in the presence of the Mfn2 gene; control (MEFwt) and with *Mfn2* gene depleted MEF^Mfn2-/-^. These two cell lines were compared in terms of various parameters characterizing mitochondrial bioenergetics. Here, we have shown that relative rate of proliferation of MEF^Mfn2-/-^ cells versus control fibroblasts depend on serum supplementation of the growth media. Moreover, MEF^Mfn2-/-^ cells exhibited significantly increased respiration rate in comparison to MEFwt, regardless of serum supplementation of the medium. This effect was correlated with increased level of mitochondrial markers (TOM20 and NAO) as well as mitochondrial transcription factor A (TFAM) and peroxisome proliferator-activated receptor gamma coactivator 1-alpha (PGC-1α) protein levels and unchanged total ATP content. Interestingly, mitochondrial DNA content in MEF^Mfn2-/-^ cells was not reduced. Fundamentally, these results are in contrast to a commonly accepted belief that mitofusin 2 deficiency inevitably results in debilitation of mitochondrial energy metabolism. However, we suggest a balance between negative metabolic consequences of mitofusin 2 deficiency and adaptive processes exemplified by increased level of PGC-1α and TFAM transcription factor which prevent an excessive depletion of mtDNA and severe impairment of cell metabolism.

## Introduction

Mitofusin (Mfn2) is encoded by nuclear DNA. It locates mainly in the outer mitochondrial membrane, and is involved in its rearrangement [1]. Moreover, Mfn2 is also present in the endoplasmic reticulum membrane and plays a role in ER interactions with mitochondria [2]. Growing interest of Mfn2 as a protein putatively involved in cell survival, begun when changes in Mfn2 expression in muscles of diabetic patients [3] and in blood vessels of patients suffering from vascular proliferative disorders [4] were observed. Moreover, a significant point in studies of mitofusin 2 is to understand the link between *MFN2* mutations and Charcot-Marie-Tooth type 2A axonal neuropathy development [5]. Mitofusin 2 is engaged in the proper formation of mitochondrial network. Fusion of mitochondria might be regarded as a way to equal distribution of mtDNA in the cells and, as a consequence, proper formation of mitochondrial complexes [6]. There are conflicting data on the composition and activity of respiratory complexes and ATP level in various tissues from CMT patients and experimental models *in vitro*. Vielhaber et al. (2012) showed a slight decrease in the activity of the respiratory complex I in fibroblasts and in the muscle fibers biopsies from CMT2A patients [7]. Earlier it was shown that mitochondrial respiratory enzyme activity was normal in skeletal muscle mitochondria from a CMT2 patient with an *MFN2* mutation and ATP production was also unaffected [8]. Finding the molecular mechanism of neuronal mitochondria metabolism and axonal loss observed in CMT2A in the absence of fully functional MFN2 would be crucial for CMT2A counseling and also desirable for therapeutic strategies of wide range of disorders. Therefore, investigations of cellular energy metabolism in mitofusin 2-depleted cells should be continued to clarify various aspects of its improper functions.

Here, energy metabolism and proliferation rate of mitofusin 2-depleted MEF cells and their mitofusin 2-positive equivalents were in focus. Relatively increased rate of oxygen consumption together with increased level of mitochondrial markers, peroxisome proliferator-activated receptor gamma coactivator 1-alpha (PGC-1α) and mitochondrial transcription factor A (TFAM) proteins and mitochondrial mass but unaffected mtDNA content suggest an increased mitochondrial biogenesis which may efficiently counteract but not outbalance detrimental effects of mitofusin 2 deficiency.

## Materials and Methods

### Cell culture

Mouse Embryonic Fibroblasts: Wild type MEFs (MEFwt) (ATCC-CRL-2991), Mfn2-null MEF (MEF^Mfn2-/-^) (ATCC-CRL-2993) were cultured in high glucose Dulbecco's Modified Eagle Medium (DMEM, Gibco) supplemented with either 10% Fetal Bovine Serum (Gibco) or 10% Bovine Calf Serum (Gibco) and antibiotics (100 units/ml penicillin plus streptomycin 100 μg/ml), in 5% CO_2_ atmosphere at 37°C.

### Cell proliferation assay

Cell doubling time was analyzed using xCELLigence RTCA DP instrument (ACEA Biosystems), which was placed in a humidified incubator at 37°C with 5% CO_2_. The electronic sensors provided a continuous and quantitative measurement of cell index in each well. Cell index represents a quantitative measure of cell number present in a well e.g. low cell index reflects fewer cells attached to the electrodes. Approximately 10 x 10^3^ of MEFs were plated into E-plate 16 and doubling time of cells were analysed based on real time measurements carried out for 48 hours [9,10].

### Cellular respiration

Cellular respiration was measured polarographically, with the use of Oxygraph-2k (OROBOROS; INSTRUMENTS GmbH, Austria). Cells from 10 cm diameter tissue culture dish were trypsinized and resuspended in pre-warmed PBS to final protein concentration 0.15–0.3 mg/ml. Oxygen consumption was measured at 37°C in substrate free solution (PBS) and after administration of 1 mM pyruvate, 5 mM glucose, 0.1 mg/ml oligomycin and CCCP (1 μM). After respiration assay, cell suspension was mixed with 0.5 M NaOH (1:1) and protein concentration was measured. Data were presented as mean use of O_2_ picomoles per milligram of protein per second.

### Mitochondrial membrane potential

Mitochondrial potential was measured fluorimetrically according to the Cossarizza et al. (2001) [11]. Cells were trypsinized, incubated with 5 μM JC-1 dye (5,5’,6,6-tetrachloro-1,1’,3,3-tetraethylbenzimidazolylcarbocyanine iodide; Molecular Probes, Invitrogen) at 37°C in the dark for 15 min. Parallel to JC-1 cell also were labeled with the nuclear stain TO-PRO-3 (0.4 μM). When indicated, prior to JC-1/ TO-PRO-3 staining, cells were preincubated for 10 min with 3 μM valinomycin and 5 μM CCCP or 0.1 μg/ml oligomycin. Fluorescence was measured by BD FACSCalibur flow cytometry equipped with argon laser (excitation 488 nm for JC-1) and excitation/emission (nm) 642⁄661 for TO-PRO-3. JC-1 fluorescence was measured only in TO-PRO-3 negative cells.

### Blue Native sample preparation, electrophoresis and in-gel complex V activity assay

MEFwt and MEF^Mfn2-/-^ cells were trypsinized, washed with cold PBS and homogenized in homogenization buffer (10 mM Tris-HCl, 0.25 M sucrose, 2 mM EDTA, 1 mM PMSF, Protease Inhibitor Cocktail from Sigma; pH 7.4) on ice. Homogenates were centrifuged at 1000 x g for 5 min. at 4°C. Supernatants were spun at 10 000 x g for 20 min at 4°C, to obtain pellet enriched in mitochondria. Samples containing 100 μg protein were pelleted and resuspended in 10 μl aminocaproic acid buffer (1.5 M ACA, 50 mM Bis-Tris; pH 7.0) with PMSF and protease inhibitor cocktail (Sigma). 1 μl of 10% dodecylomaltoside was added to each sample. After 30 min incubation on ice, samples were spun at 17 000 x g for 20 min at 4°C. Supernatants were mixed with 0.4 μl aminocaproic acid buffer with 5% Coomassie G-250 (BioRad) and loaded onto 5–12% gradient acrylamide gel. Electrophoresis was started with 70 V in cathode buffer (50 mM Tricine, 15 mM Bis-Tris/HCl, pH 7.0 at 4°C with 0.02% Coomassie G-250) and anode buffer (50 mM Bis-Tris/HCl, pH 7.0 at 4°C). After 1 h, voltage was increased to 200 V. To visualize complex V activity, gel stripes were incubated over night at room temperature in solution containing 35 mM Tris HCl, 270 mM glycine (pH 8.3–8.6), 14 mM MgCl_2_, 0.1% Pb(NO_3_)_2_, 8 mM ATP according to Sabar et al., 2005 and Yan et al., 2009 [12,13]. An intensity of bands corresponds to ATP-ase enzymatic activity.

### Western blot analysis

(WB) of selected respiratory chain proteins and mitochondrial ATP synthase subunit were performed with MitoProfile Total OXPHOS Rodent WB Antibody Cocktail (MitoScience). Cell extracts were prepared with Lysis Buffer (Cell Signalling), separated by 12% SDS-polyacrylamide gel electrophoresis (30 μg of protein per line) and electro-transferred to nitrocellulose membrane. In WB, following subunits were detected: NDUFB8 (complex I), CII-30 kDa (FeS; complex II); CIII-Core 2 protein (complex III); C-IV-I subunit (complex IV); C-V-alpha subunit (complex V). Data were expressed as relative immunoreactivity of each complex in knock-out cells in relation to wild type one. To study the mitochondrial biogenesis the following primary antibodies were used: TFAM (GenWay Biotech, GWB-22C6C2), TOM20 (Santa Cruz Biotechnology, sc-11415), PGC-1α and PCNA, β-actin and GAPDH as loading markers (Santa Cruz Biotechnology, sc-13067, sc-9857, Sigma-Aldrich, A2066 and EMD Millipore, MAB374, respectively).

### Mitochondrial mass with NAO

Cells were trypsynized and suspended in the fresh culture medium supplemented with 1 μM NAO, incubated in 37°C in the atmosphere of 5% CO_2_/95% air for 30 minutes, according to [14]. After washing the cells were suspended in PBS with calcium and magnesium ions. Measurements were done in FACS CAlibur with excitation/emission 495/522 nm. In each sample 10 000 events were counted. The data are expressed as a geometric mean of fluorescence ± SD from 6 independent experiments.

### ATP content

ATP was measured enzymatically as described earlier [15], according to the method of Williamson and Corkey (1969) [16]. Cells were incubated in Krebs—Henseleit solution (10 mM HEPES, 2 mM NaHCO_3_, 135 mM NaCl, 3.5 mM KCl, 0.5 mM NaH_2_PO_4_, 0.5 mM MgSO_4_, 1.5 mM CaCl_2_, 1 mM pyruvate, pH 7.4) without or with glucose (5.6 mM), ATP synthase inhibitor oligomycin (0.1 μg/ml) or glycolysis inhibitor iodoacetate (1 mM) for 10 min at 37°C. Then the incubation solution was removed and cells were extracted with 3.5% HClO_4_ for 10 min on ice. Extracts were collected and neutralized with 2 M K_2_CO_3_. ATP concentration was measured fluorimetrically, with the use of glucose-6-phosphate dehydrogenase and hexokinase in pH 7.4 buffer (50 mM triethanolamine-HCl, 10 mM MgCl_2_, 5 mM EDTA). ATP concentration was standardized with the use of ATP solution of known concentration. Protein concentration was measured in cells solubilized in 0.5 M NaOH. Data were presented as ATP concentration (nanomoles per milligram of protein) ± S.D. for 3–5 independent experiments.

### Lactate synthesis

Lactate synthesis was estimated on a basis of enzymatic measurement of lactate concentration in the acidified incubation medium, according to [17]. It allowed determining both intracellular lactate content and lactate released from cells. The cells were incubated in Krebs—Henseleit solution (10 mM HEPES, 2 mM NaHCO_3_, 135 mM NaCl, 3.5 mM KCl, 0.5 mM NaH_2_PO_4_, 0.5 mM MgSO_4_, 1.5 mM CaCl_2_, supplemented with 1 mM pyruvate and 5.6 mM glucose, pH 7.4) for 30 min. Next, the incubation was acidified with HClO_4_ (final concentration 5%). After 10 minutes on ice extracts were neutralized with 2 M K_2_CO_3_ and lactate was measured fluorimetrically, with the use of lactate dehydrogenase in a buffer composed of 0.5 M glycine, 0.4 M hydrazine sulphate, 5 mM EDTA pH 9.5. Lactate concentration was calculated with the use of standardized lactate solution. Protein concentration was measured in cells solubilized in 0.5 M NaOH. Data were presented as lactate content (nanomoles per milligram of protein) ± S.D. for 6 independent experiments.

### mtDNA content

mtDNA content was measured by real-time PCR, according to [18]. Quantification was based on mtDNA to nuclear thymidylate kinase gene ratio [19]. Total DNA was extracted by Genomic Mini Kit (A&A Biotechnology). PCR amplifications were carried out with SYBR Select Master Mix (Applied Biosystems) on Applied Biosystems 7500 real-time PCR thermal cycler in conditions: 50°C for 2 min and 95°C for 10 min followed by 40 cycles of 95°C for 15 s and 60°C for 1 min. Following primers were used: Mouse mitochondrial displacement loop (D-loop) region (D-loop F, 5’-CCAAAAAACACTAAGAACTTGAAAGACA-3’ and D-loop R, 5’-GTCA TATTTTGGGAACTACTAGAATTGATC-3’) and single-copy nuclear thymidylate kinase gene (F, 5’-GACTGTATTGAGCGGCTTCAGA-3’ and R, 5’-CATGCTCGGTGTGAGCCATA-3’). Relative mtDNA quantity was determined using ΔΔCt method, according to Livak and Schmittgen (2001) [19].

### Protein concentration

Protein was measured with the use of Modified Lowry Protein Assay Kit (Pierce).

### Statistics

Data are shown as mean value ± standard deviation. The statistical significant differences were calculated using Bonferroni or Student’s t-est for the simultaneous analysis of multiple test groups preceded by analysis of variance ANOVA.

## Results

### Cell proliferation

**Fig 1:**
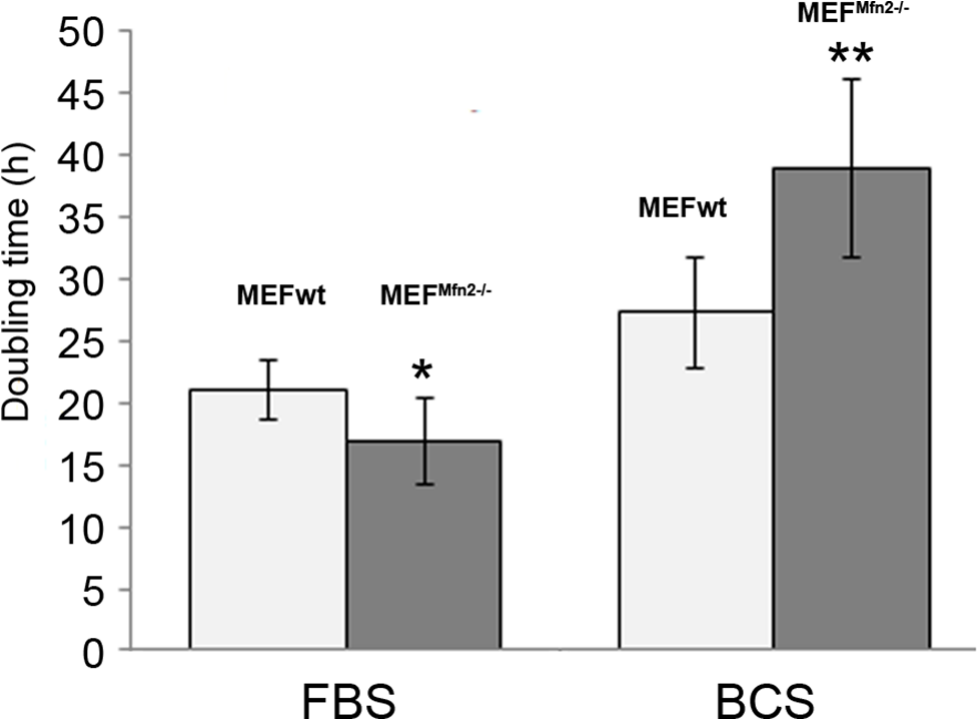
Doubling time of MEFwt and MEF^Mfn2-/-^ cells. The cells were grown in media supplemented with either FBS or BCS. Doubling time of cells were analysed based on real time measurements carried out for 48 hours using xCELLigence RTCA DP instrument. Data show mean values ± S.D. n = 10 * p < 0.05, ** p < 0.01.

Mitofusin-deficient cells were previously found to proliferate faster that their mitofusin 2-positive equivalents and this was attributed to the mitofusin 2-dependent inhibition of the Ras-Raf-ERK signaling pathway [20]. However, in view of inconsistency concerning type of serum for supplementation of the growth media (FBS is commonly used by researchers and BCS is recommended by supplier) which may imply different composition of growth factors, the rate of both cell lines proliferation in the presence of either FBS or BCS in the medium was here determined. It was found that MEF^Mfn2-/-^ cells proliferated much faster than MEFwt cells when grown in the FBS supplemented medium while in BCS it was completely reversed; proliferation of the mitofusin 2-deficient cells was substantially slowed-down in comparison to proliferation of the mitofusin–positive fibroblasts. Interestingly, proliferation of the latter was less sensitive to the type of serum (Fig 1).

### Oxygen consumption and mitochondrial membrane potential

**Fig 2:**
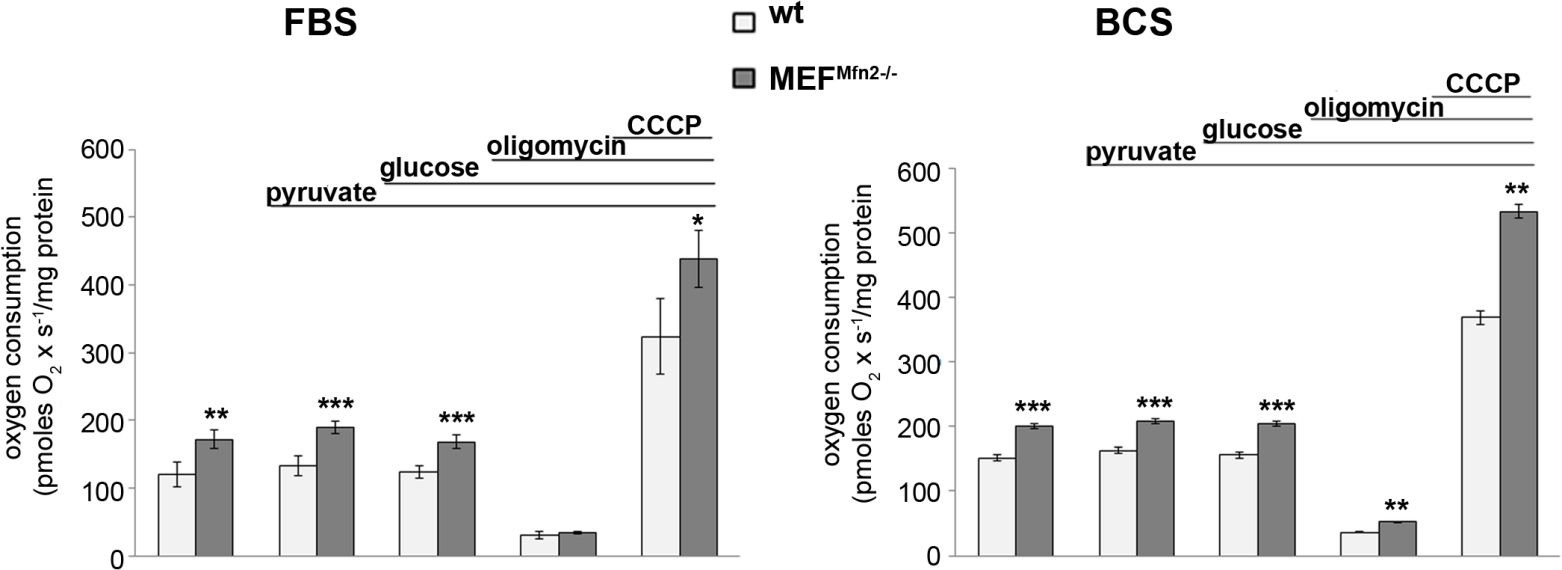
Oxygen consumption by MEFwt and MEF^Mfn2-/-^ cells. The cells were grown in media supplemented with either FBS or BCS. Oxygen consumption was measured polarographically at 37°C in a substrate free buffered saline solution (PBS with Mg^2+^ and Ca^2+^), and after sequential administration of 1 mM pyruvate, 5 mM glucose, 0.1 mg/ml oligomycin and 1 μM CCCP. Data are expressed as pmoles O_2_ x s^-1^/mg protein and show mean values ± S.D. n = 4; *p<0.01, **p<0.001, ***p<0.0001.

Rate of oxygen consumption is a complex measure of cellular energy metabolism. It depends on a variety of parameters such as substrate and oxygen availability, cellular energy charge, activity of respiratory complexes and mitochondrial membrane potential. A comparison of mitochondrial respiration in various cells under different experimental conditions allows concluding about differences in their metabolic capability. This is a first test typically performed to characterize bioenergetics state of cells. Here two lines of mouse embryonic fibroblasts MEF have been investigated. They were MEF^Mfn2-/-^ and their wild type equivalent (MEFwt) as a control. As shown in Fig 2, MEF cells with deleted *Mfn2* gene exhibit substantially faster oxygen consumption than control fibroblasts.

This statistically significant difference is very similar in cells incubated without any exogenous substrates as well as in the presence of glucose and pyruvate. It indicates that availability of endogenously stored respiratory substrates does not limit respiration rate in both cell lines tested, thus it cannot be responsible for observed differences. Moreover, the same tendency was found in cells treated with mitochondrial uncoupler. The latter may suggests an increased cellular respiratory capacity presumably due to elevated content of mitochondrial respiratory enzymes, enhanced biogenesis of these organelles or increased enzymatic capacity of respiratory complexes. Indeed, respiratory capacity expressed as a ratio between rate of oxygen consumption in the presence of CCCP to that in the presence oligomycin prior to mitochondrial uncoupling was very similar in both MEFwt and MEF^Mfn2-/-^ cells (Table 1) suggesting unaffected specific enzymatic activity of the respiratory chain.

**Table 1:** Respiratory capacity of MEFwt and MEF^Mfn2-/-^ cells.

FBS		BCS	
MEFwt	MEF^Mfn2-/-^	MEFwt	MEF^Mfn2-/-^
9.90 ± 069	10.01 ± 04.6	10.5 ± 0.66	10.24 ± 0.51

The cells were grown in media supplemented with either FBS or BCS, as indicated. Respiratory capacity was expressed as a ratio between maximal (upon addition of CCCP) and minimal (in the presence of oligomycin prior to addition CCCP) rates of oxygen consumption. Results represent mean values of the ratio ± S.D. n = 4.

**Fig 3:**
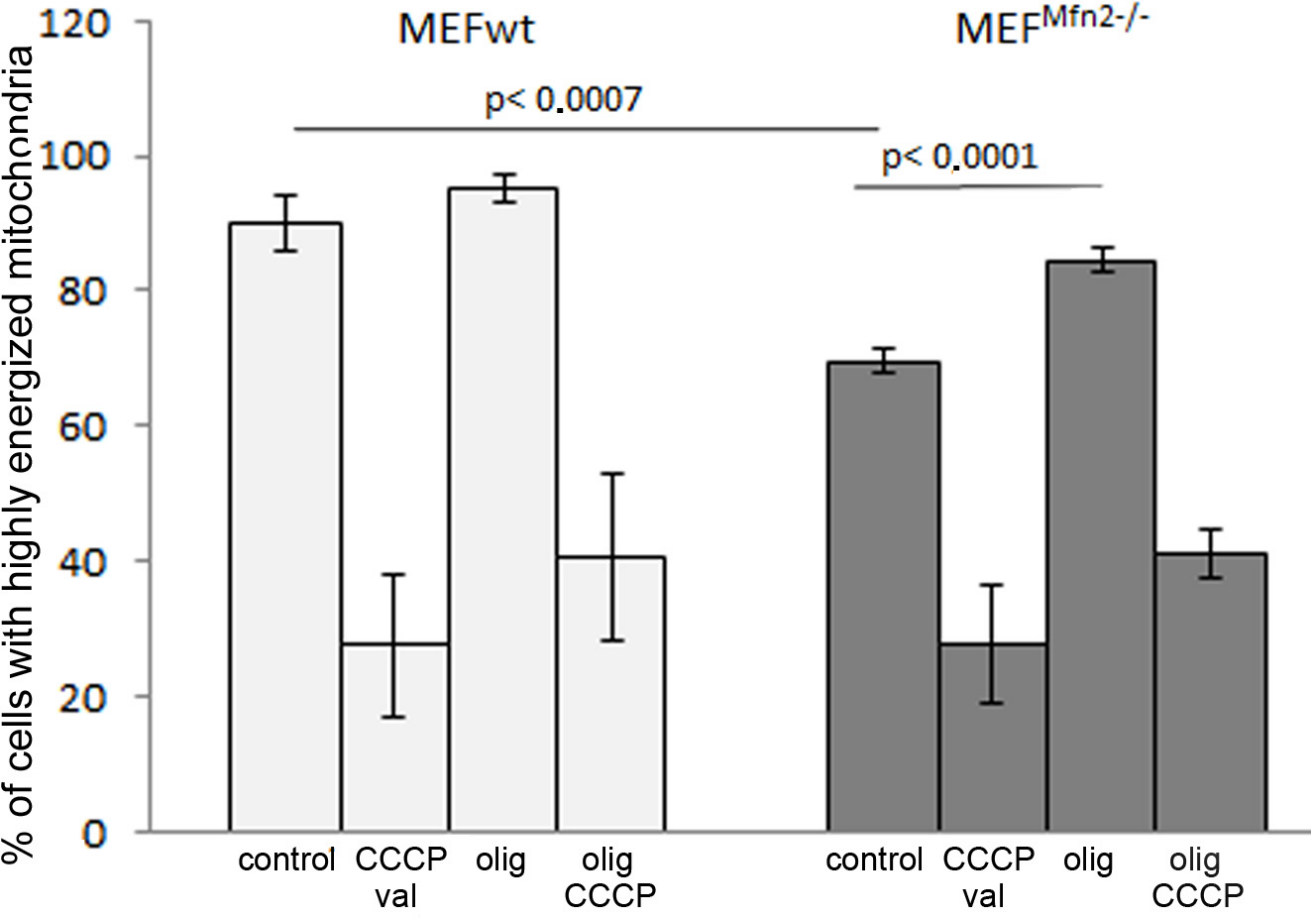
Mitochondrial membrane potential in MEFwt and MEF^Mfn2-/-^ cells. The cells were grown in the medium supplemented with FBS. Mitochondrial membrane potential was determined fluorimetrically with JC-1 probe using flow cytometry. JC-1 fluorescence was measured in TO-PRO-3 negative cells (more than 95% of total population), which were assumed as 100%. Ordinate shows a proportion of cells with energized mitochondria. Data show mean values ± S.D. n = 6.

**Fig 4:**
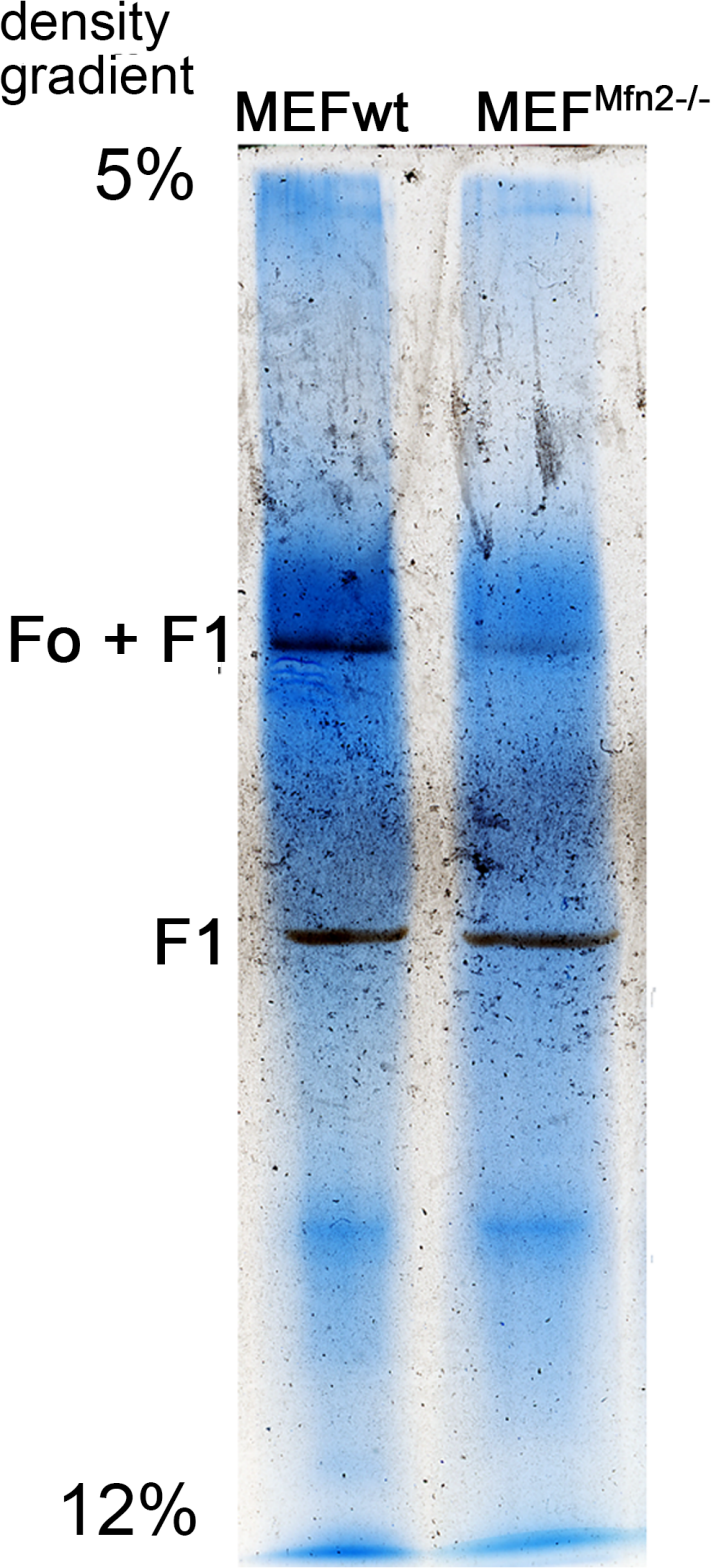
Effect of mitofusin deficiency on ATP-ase (complex V) activity. The cells were grown in the medium supplemented with FBS. In-gel activity of entire F1Fo complex and separated F1 subunit in MEFwt and MEF^Mfn2-/-^ cells were measured after blue-native polyacrylamide gel electrophoresis. One representative image out of three independent experiments is shown.

More intense oxygen consumption by cells depleted of mitofusin 2 and unaffected respiratory capacity were observed irrespectively of a type of serum supplementing the growth media (Table 1). To exclude cell-line specific effects, the experiments were repeated in a second set of MEFwt and MEF^Mfn2-/-^ cells purchased from the same supplier but differing in their batch number, and the same responses were found. Thus a medium-dependent changes of MEF^Mfn2-/-^ proliferation seems to be unmatched with faster respiration of mitofusin-deficient cells. High consistency of obtained results excluded randomness of our observations. In further experiments cells grown in the FBS-supplemented medium were used only. Faster oxygen consumption by MEF^Mfn2-/-^ cells, especially in the presence of mitochondrial uncoupler suggests more active respiratory chain. However it does not exclude coexistent lower mitochondrial membrane potential, which also could explain increased respiration rate prior to addition of CCCP. Indeed, as shown in Fig 3, a treatment of MEF^Mfn2-/-^ fibroblasts with oligomycin substantially increases a proportion of cells with high mitochondrial membrane potential (ΔΨ) while in the mitofusin 2-positive cells, mitochondrial potential is higher and likely it makes them less sensitive to oligomycin. Oligomycin inhibits mitochondrial ATPase, thus it seems clear that reduced mitochondrial membrane potential in mitofusin 2-deficient fibroblasts is not due to increased unspecific or generalized permeability of mitochondrial membrane for protons but rather reflects selective oligomycin-sensitive permeability of ATPase for these cations. As shown in the Fig 4 in-gel activity of complex V is identified in two bands corresponding to entire Fo-F1 complex and separated catalytic F1 subunit. Interestingly, in a case of MEF^Mfn2-/-^ cells a proportion of the latter is significantly higher than in the control cells. This may indicate disrupted stoichiometry between particular subunits composing complex V leading to disability to complete assembling Fo-F1 subunits and presumably increased oligomycin-inhibitable proton permeability through Fo subunits not assembled with F1, leading to dissipation of the mitochondrial membrane potential. However, it does not exclude partial contribution of ATP synthesis in a reduction of ΔΨ, which is prevented by oligomycin.

### Mitochondrial mass, transcription factors and respiratory chain complexes

**Fig 5:**
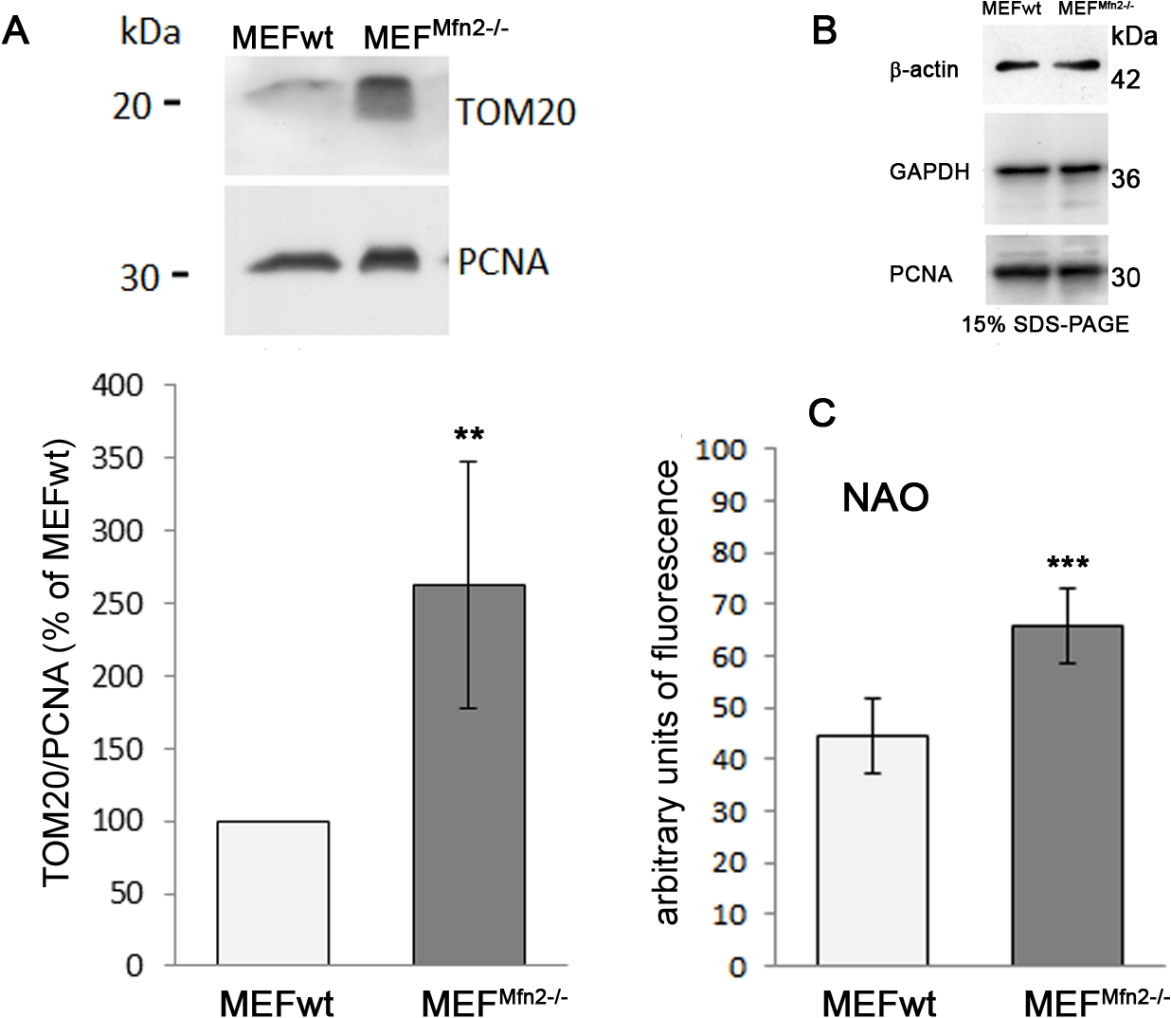
Effect of mitofusin deficiency on the amount of TOM20 protein and mitochondrial mass in MEF cells. The cells were grown in the medium supplemented with FBS. (A). Relative amount of mitochondrial marker TOM20 in MEFwt and MEF^Mfn2-/-^ cells were tested immunochemically by Western blotting and normalized to immunoreactivity of PCNA. Representative blot as well as mean values of immunoreactivity ± S.D. for TOM20 are shown. Immunoreactivity of this protein in wild type cells is assumed as 100%, n = 4, **p<0.001. (B) Comparison of three loading markers (PCNA, β-actin and GAPDH) level in MEFwt and MEF^Mfn2-/-^ cells. There are no differences in the immunoreactivity between these proteins in both cell lines tested. (C) Mitochondrial mass was estimated by NAO staining of cardiolipin. Fluorescence was measured with flow cytometry. n = 6, *** p < 0.0005.

**Fig 6:**
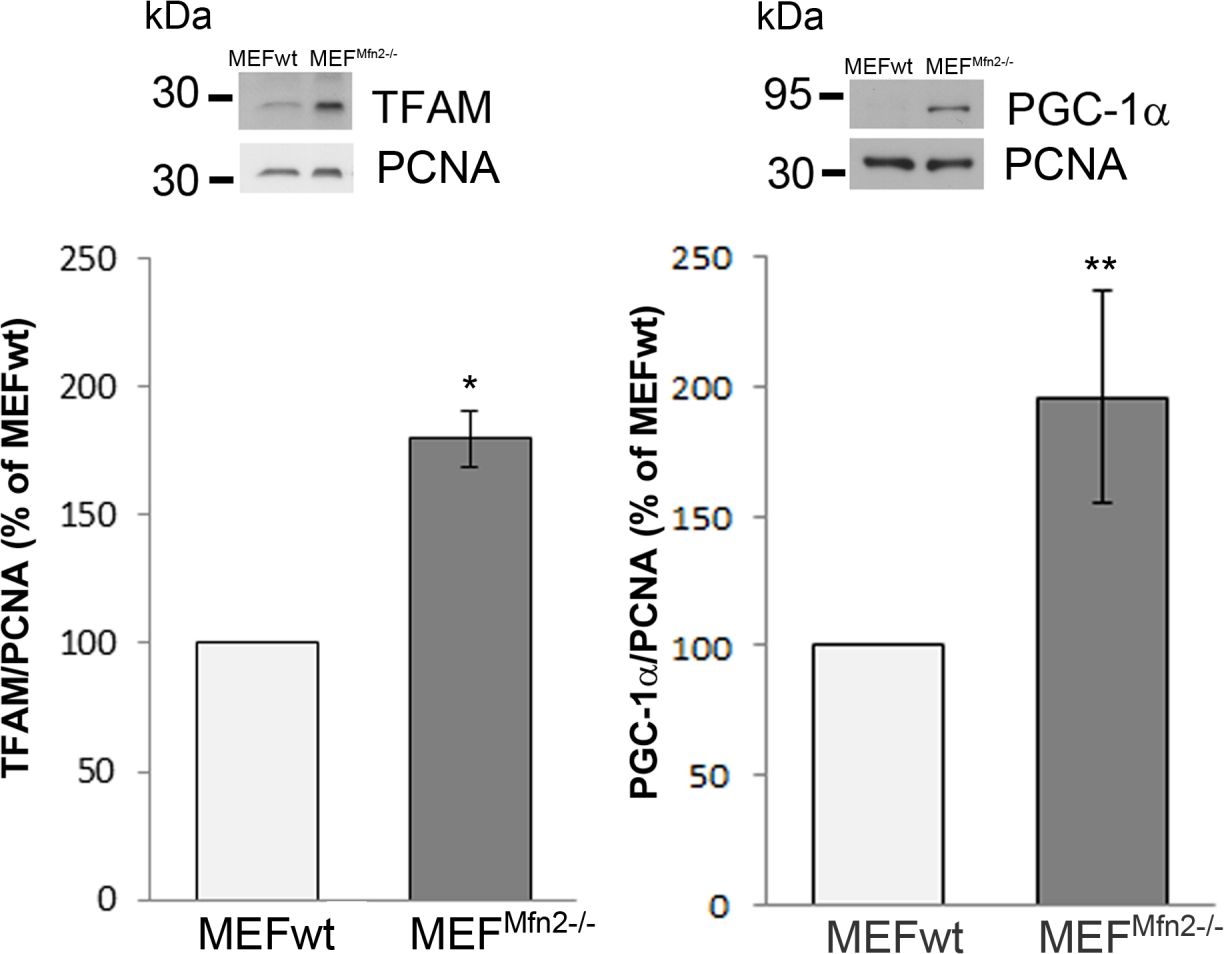
Effect of mitofusin deficiency on the amount of TFAM and PGC-1α protein in MEF cells. The cells were grown in the medium supplemented with FBS. Relative amounts of both proteins in MEFwt and MEF^Mfn2-/-^ cells were tested immunochemically by Western blotting and normalized to immunoreactivity of PCNA. Representative blots and mean values of immunoreactivity ± S.D. are shown. Immunoreactivity of these proteins in wild type cells is assumed as 100%, n = 4, *p<0.01. ** p<0.05.

**Fig 7:**
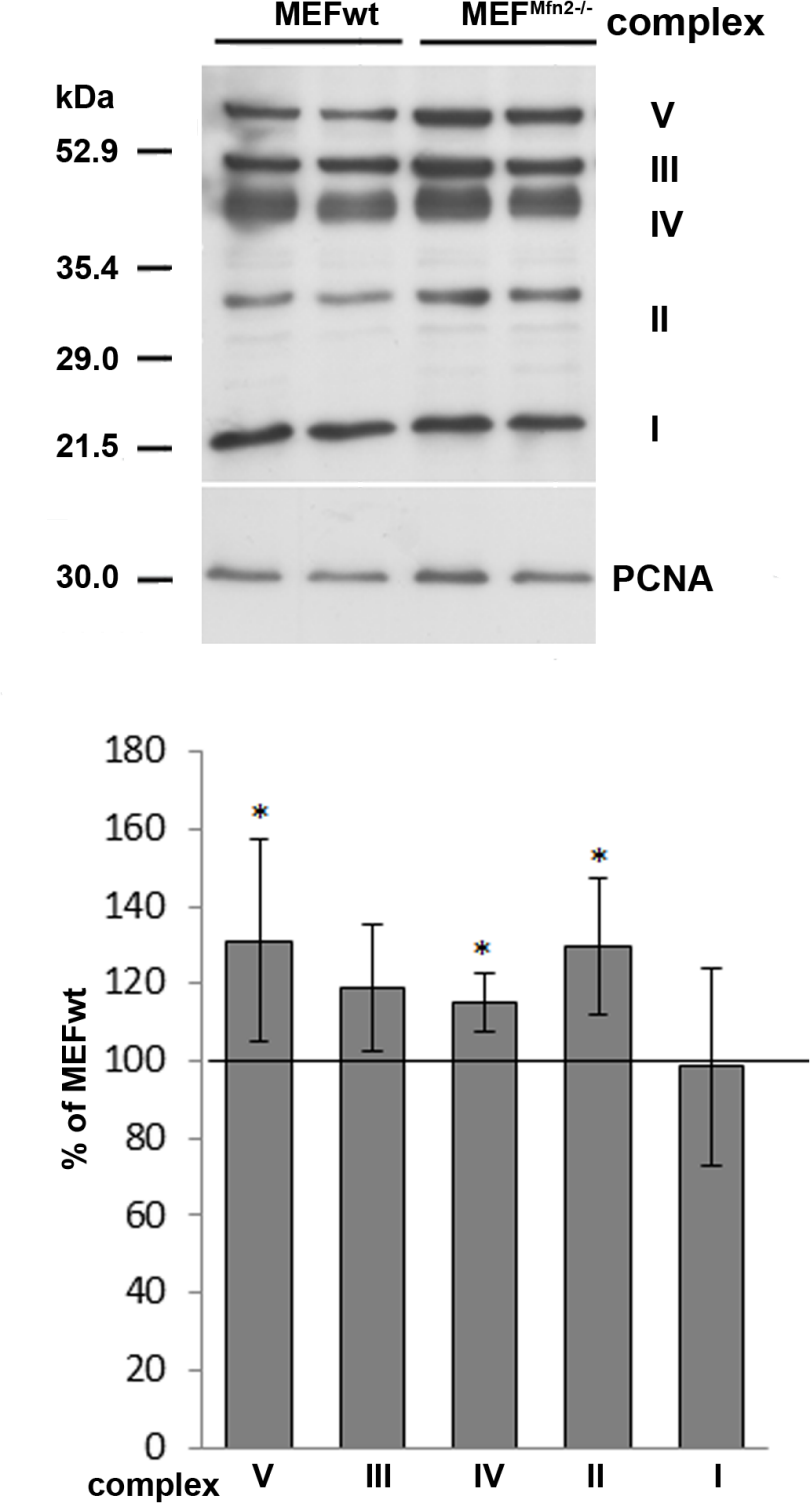
Effect of mitofusin deficiency on respiratory complexes protein level in MEF cells. The cells were grown in the medium supplemented with FBS. Selected respiratory complexes proteins were detected in MEFwt and MEF^Mfn2-/-^ cells by Western blotting. Representative blot of selected mitochondrial complexes subunits and densitometric analysis of at least six independent experiments are shown. Data are presented as mean immunoreactivity normalized to immunoreactivity of PCNA ± S.D. Immunoreactivity of these proteins in wild type cells is assumed as 100%, n = 6–8, *p<0.05.

Aforementioned suggestion that MEF^Mfn2-/-^ fibroblasts may have more mitochondria (greater mitochondrial mass) than MEFwt cells was supported by Western blotting indicating that *Mfn2*-depleted cells exhibit significantly higher level of TOM20 which is an accepted mitochondrial marker protein (Fig 5A) [20]. Moreover, more intense staining of MEF^Mfn2-/-^ cells with NAO probe which binds to cardiolipin, a specific mitochondrial inner membrane phospholipid (Fig 5B) enforces this hypothesis. Further confirmation of this suggestion comes from Western blot analysis of TFAM which is a mitochondrial transcription factor and a key activator of mitochondrial DNA replication [21]. As shown in Fig 6, TFAM level in MEF^Mfn2-/-^ cells is much higher than in MEFwt fibroblasts. Furthermore, the same was observed when PGC-1α protein level was analyzed (Fig 6). Both proteins are involved in mitochondrial biogenesis and increased level of them is considered as a marker of increased rate of this process [22]. Thus, these data convincingly indicate more intense “mitochondriogenesis” which may compensate some mitochondrial dysfunction shown hitherto and described as a consequences of mtDNA copy number reduction by others [7]. In line with this assumption, Western blot analysis of selected subunits of the mitochondrial respiratory chain complexes has revealed reproducibly higher level of complexes III, IV and V in mitofusin 2-depleted fibroblasts than in the MEFwt cells (Fig 7). Interestingly, respiratory complex I level seems to be the same in both cell lines tested. This may suggest its relatively low impact on the metabolic control of the respiratory chain in mitofusin 2-positive fibroblasts. It is also possible that slightly but reproducibly increased ROS formation in mitofusin 2-deficient cells (data not shown) could reflect relatively higher rate-controlling participation of the complex I to the electron flow than in mitofusin 2-positive cells. Increased amount of the complex V (ATPase) in MEF^Mfn2-/-^ cells probably counterbalances, improper composition of this protein complex. Altogether, data shown here indicate that mitochondrial metabolism of MEF cells with depleted mitofusin 2-encoding gene differs from that found in the wild type MEF and these change may be attributed to adaptations reflected by enhanced mitochondrial biogenesis.

### ATP content and lactate formation

**Fig 8:**
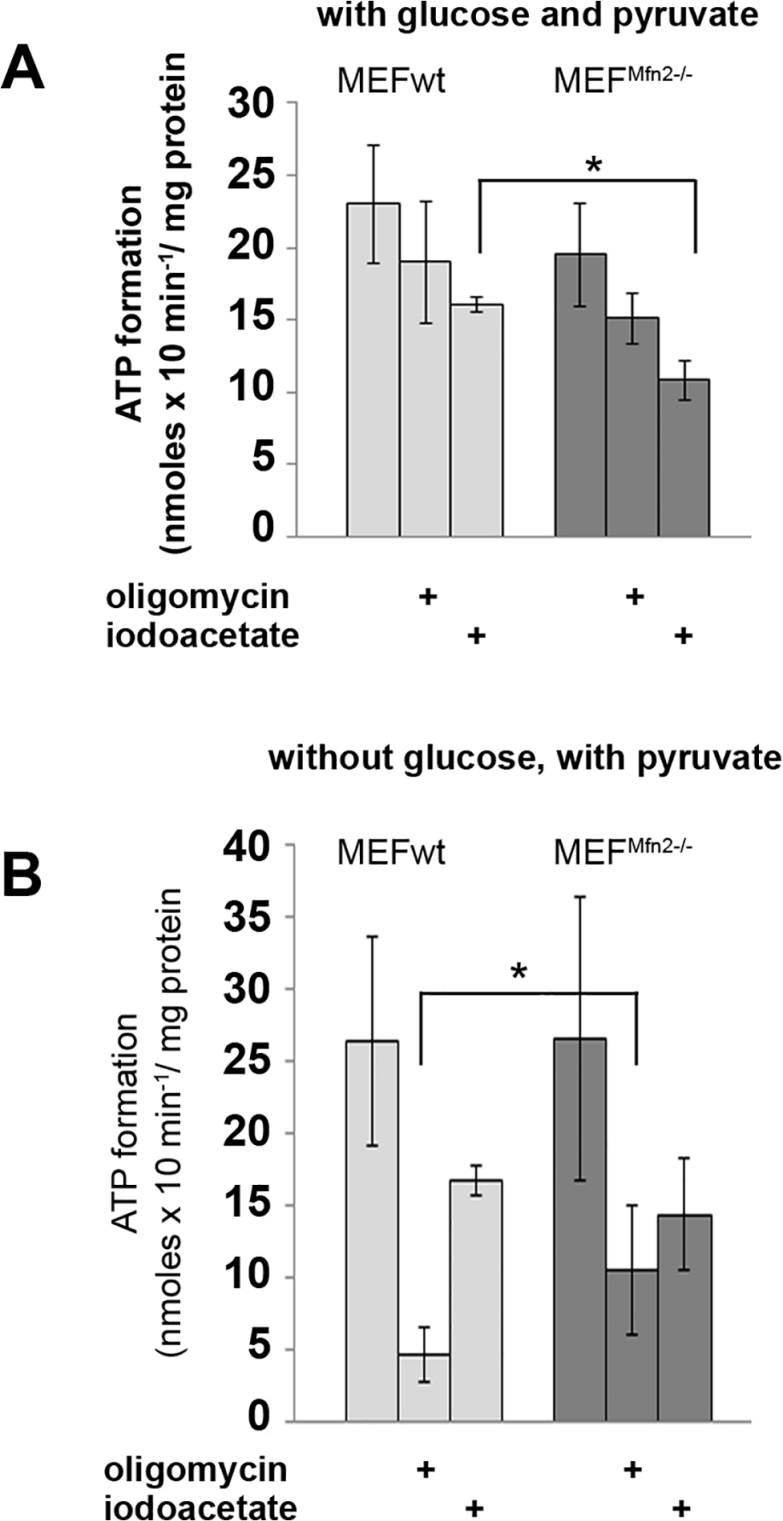
Effect of mitofusin 2 deficiency on ATP content in MEF cells treated with oligomycin and iodoacetate. MEFwt and MEF^Mfn2-/-^ cells grown in the medium supplemented with FBS were rinsed and incubated for 10 min in the presence of 1 mM pyruvate and 5.6 mM glucose (A) or in glucose-free Krebs-Henseleit solution (B). ATP was measured in neutralized acidic cell extracts. Where indicated the cells were treated with oligomycin (0.1 μg/ml) and iodoacetate (1 mM) to inhibit oxidative phosphorylation and glycolysis, respectively. Data are expressed as a mean amount of ATP (nanomoles per milligram of protein) ± S.D.; n = 3–5 * p < 0.05.

Difference in mitochondrial metabolism could also influence an efficiency of the oxidative phosphorylation. As shown in Fig 8 ATP content in MEF^Mfn2-/-^ cells seems to be less sensitive to oligomycin (particularly in the absence of glucose in the assay–Fig 8B) and more sensitive to iodoacetate (especially in the presence of glucose–Fig 8A) than in the MEFwt fibroblasts. These differences are rather minor and indicate only slight switch from aerobic to anaerobic ADP phosphorylation in *Mfn2*-deficient fibroblasts. Similarly in the presence of 5.6 mM glucose in the extracellular milieu the absolute amount of ATP in untreated cells (control bars in the Fig 8) after 10 min of incubation are very similar (nmol/mg protein, n = 5): 23.0 ± 4.0 and 19.5 ± 3.5 for MEFwt and MEF^Mfn2-/-^ cells, respectively. Table 2 shows relative effects of oligomycin and iodoacetate on ATP level. MEFwt incubated without glucose are particularly sensitive to ATPase inhibition, indicating more significant dependence of these cells on aerobic metabolism than mitofusin 2-deficient cells under the same conditions.

**Table 2:** Relative effects of oligomycin and iodoacetate on ATP content in MEF cells incubated with or without glucose in the reaction buffer.

**with glucose**			
MEFwt		MEF^Mfn2-/-^	
+ olig	+ IA	+ olig	+ IA
0.82 ± 0.12 p < 0.07	0.59±0.01 p < 0.015	0.79±0.14 p < 0.008	0.45±0.03 p < 0.027
**without glucose**			
MEFwt		MEF^Mfn2-/-^	
+ olig	+ IA	+ olig	+ IA
0,21 ± 0.04 p < 0.0001	0.54 ± 0.11 p < 0.004	0.47 ± 0.18 p < 0.0008	0.46 ± 0.02 p < 0.014

The cells were grown in the medium supplemented with FBS. Data shown in the Fig 8 are presented as ratios between amount of ATP in cells incubated with oligomycin (olig) or iodoacetate (IA) and untreated cells of the same type (MEFwt_,_ or MEF^Mfn2-/-^, respectively). Amount of ATP in cells untreated with oligomycin or CCCP was assumed to be 1. Results represent mean values of the ratio ± S.D. for n = 3–5. p values were calculated using paired *t*-Student test in a relation to ATP formation rate in cells not treated with inhibitors.

**Fig 9:**
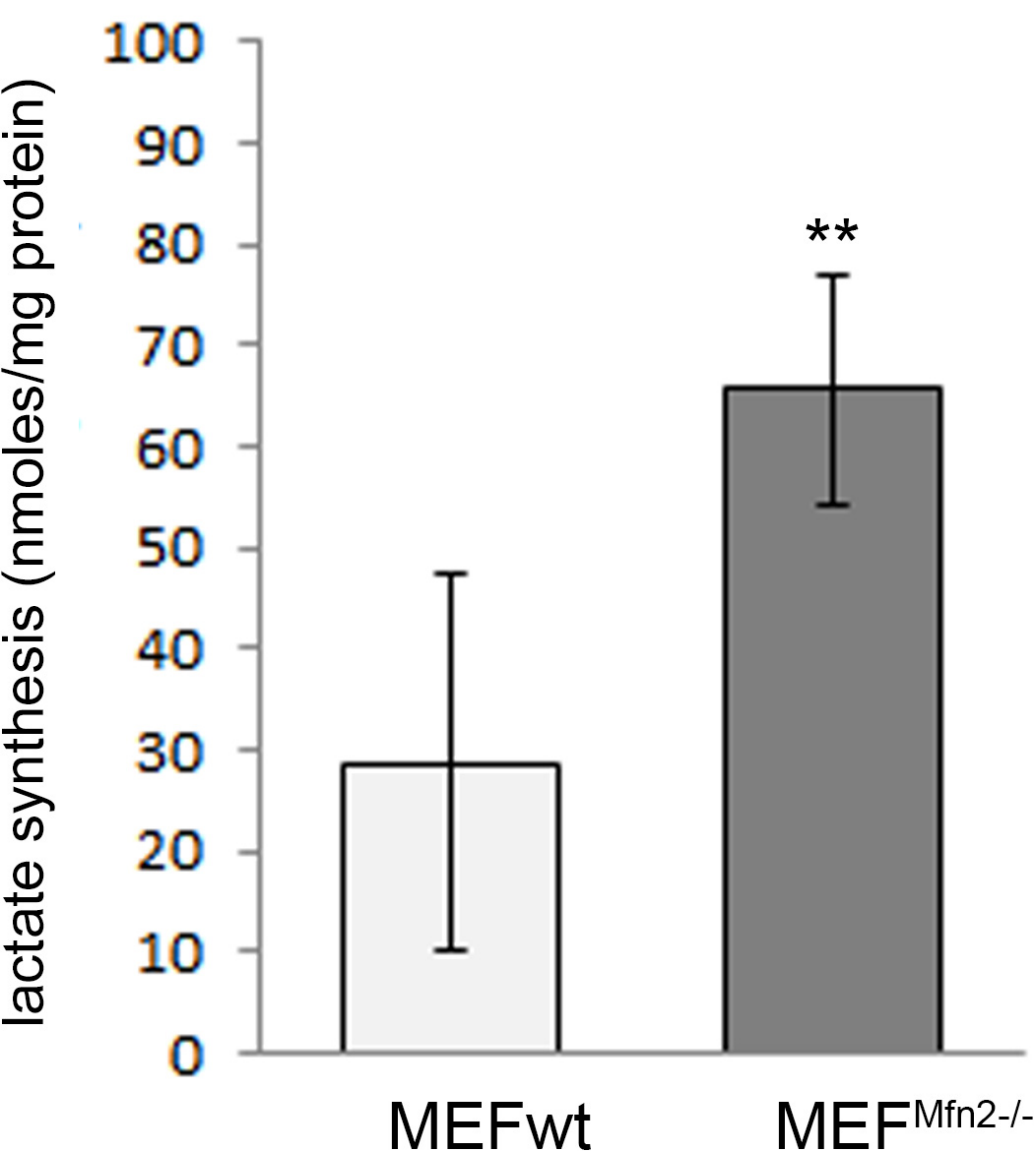
Effect of mitofusin 2 deficiency on lactate synthesis in MEF cells. MEFwt and MEF^Mfn2-/-^ cells grown in the medium supplemented with FBS. Lactate concentration was measured enzymatically in acidic extracts obtained by an addition of HClO_4_ into incubation (final concentration 5%). Data are expressed as a mean amount of lactate (nanomoles per milligram of protein) ± S.D.; n = 6, ** p < 0.003.

Substantially faster lactate synthesis, which indicate anaerobic pyruvate reduction instead of its oxidation in mitochondria in MEF^Mfn2-/-^ than in the MEFwt cells (Fig 9) supports this suggestion, convincingly.

### mtDNA content

**Fig 10:**
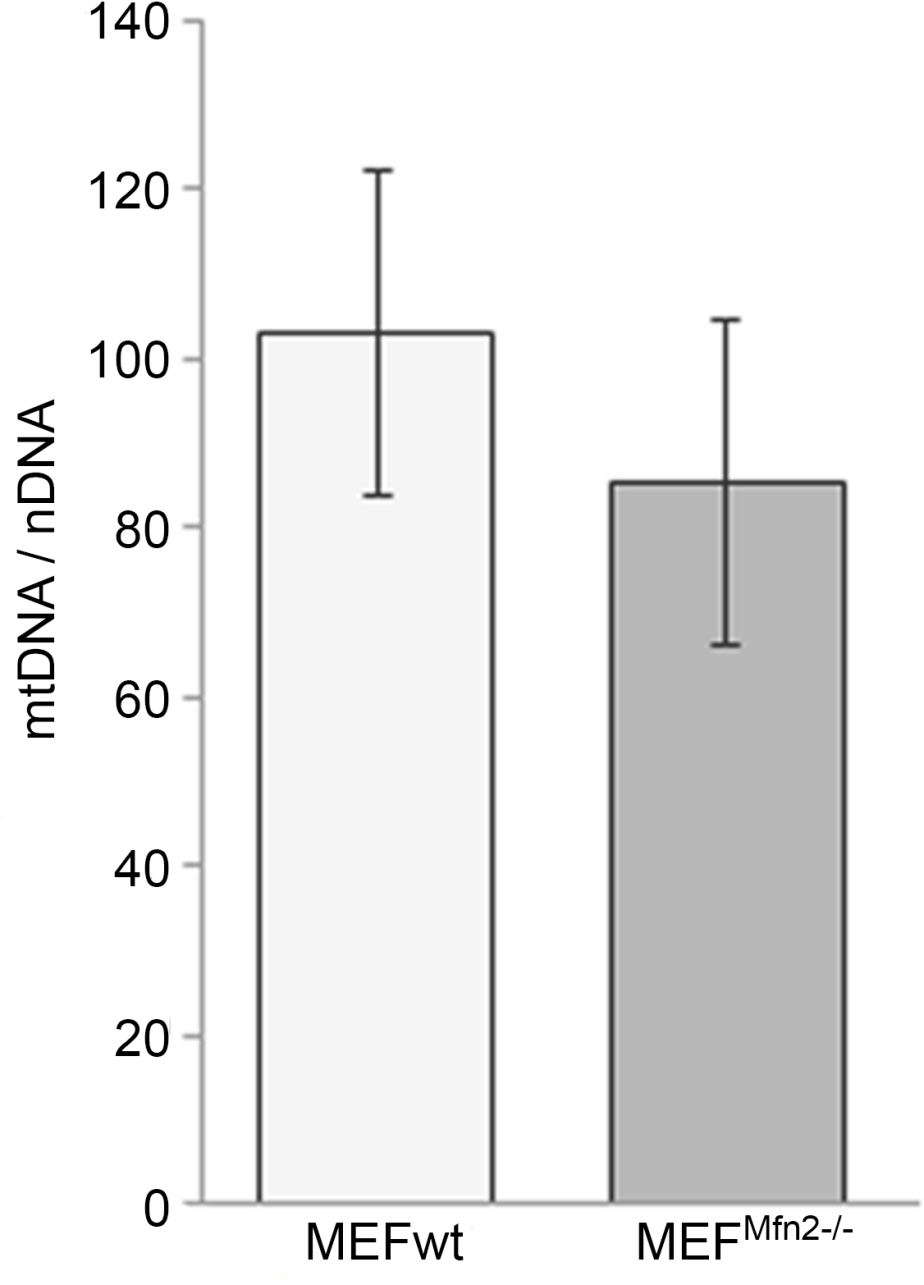
Effect of mitofusin deficiency on mtDNA content in MEF cell. Amount of mtDNA was determined in MEFwt and MEF^Mfn2-/-^ cells grown in the medium supplemented with FBS by real time PCR. Quantification was based on mtDNA to nuclear thymidylate kinase (ntDNA) gene encoding ratio. Mean values and standard deviations (indicated by error bars) were calculated from at least 6 independent experiments.

All above mentioned data convincingly indicate stimulation of mitochondrial biogenesis, increased respiration, and only slightly reduced oxidative phosphorylation. These highly coherent observations are not consistent with data previously published by others, who described reduction of mitochondrial activity attributed to reduced mitochondrial DNA content. Here examining the content of mtDNA in both cell lines, revealed slight tendency to decrease of mtDNA content in *Mfn2*-deficient cells in comparison to mtDNA in MEFwt (Fig 10) despite increased TFAM and PGC-1α protein levels. Although a source of such discrepancy is difficult to be pointed out precisely data shown here presumably reflect an adaptive effort and “rescue” response of cells which lead to a new balance between faster mtDNA degradation and enhanced mitochondrial biogenesis. It allows MEF^Mfn2-/-^ cells to survive without any severe metabolic abnormalities at least concerning ATP generation.

## Discussion

Mitofusins belong to the group of proteins responsible for proper mitochondrial network formation [23,24]. Thus, it could be assumed that their dysfunction or absence seriously affects cellular energy metabolism, calcium homeostasis and eventually many cellular functions. In fact, out-of-function mutations in *Mfn2* gene were found to reduce mitochondrial respiration and decrease selected respiratory complex subunits [25]. Similarly, liver cells derived from *Mfn2* knock-out mice exhibited reduced oxygen consumption and decreased activity of the respiratory chain complexes while respiratory rate in muscle cells of similar animals was unchanged although respiratory control was reduced [26]. Also, oxygen consumption was reduced if mitofusin 2 protein content had been reduced by the knock-down approach generated by antisense nucleotides or if mitofusin 2 was truncated by the depletion of the mitochondrial-network forming domain [25]. Moreover, since a defect of mitochondrial coupling, associated with a reduction of the mitochondrial membrane potential was observed, it was suggested that the sharply reduced efficacy of oxidative phosphorylation in *MFN2*-related CMT2A may contribute to the pathophysiology of the axonal neuropathy [27]. Mitochondrial dysfunction due to reduced mitochondrial DNA copy number, but not the impairment of mitochondrial mass or deletions in mtDNA, was also shown in three patients with new MFN2 mutations. In contrast to the copy number reduction, the deletions are unlikely to contribute to the respiratory impairment because of their minor overall amounts in the patients [7]. In contrast to aforementioned data it has been shown here that MEF cells with deleted *Mfn2* gene exhibit substantially faster oxygen consumption than control fibroblasts. The interesting effects were observed by Segales et al. (2013) [28], who found increased routine (basal) but not maximal oxygen consumption in myotubes and hepatoma FAO cells with silenced *Mfn2* gene. However, this effect was attributed to increased oligomycin-insensitive proton leak. On the other hand, the same authors shown that transfection of C2C12 cells with truncated *Mfn2* depleted of transmembrane domains and C-terminal part resulted in an enhancement of both basal and maximal mitochondrial oxygen consumption. Results shown here clearly indicate increased maximal respiration rate in the mitofusin 2-depleted cells. Parallel effect was observed in skin fibroblasts obtained from CMT2A patients harboring missense mutations of the *MFN2* gene [27]. It indicates that effects of mitofusin 2-deficiency may vary and probably depend on origin of cells tested. It has to be added that mitofusin 2 deficiency pertain to only a small part of the cases with CMT2A, while most of the cases are due to dominant mutations in the *MFN2* gene. Moreover, the neuronal specific expression of Mfn2 R94Q mutation in mouse was able to mimic the symptoms of CMT2A [29]. It is also possible that long-term adaptation in knock-out cells completely deprived of *Mfn2* gene (as used here) and thus stable deficient of mitofusin 2 protein developed adaptive mechanisms which allowed maintaining unchanged oxidative phosphorylation (OXPHOS) proteins and oxygen consumption rate. Also a compensatory effect of mitofusin 1 cannot be excluded as the oxygen consumption and OXPHOS protein content in double knockout cells are severely reduced (data not shown). It must also be noticed that ATP level in MEF^Mfn2-/-^ cells is unchanged in relation to MEFwt although relative participation of oxidative phosphorylation and glycolysis in global ATP formation is slightly shifted towards the latter. Finally, total cellular capability for ATP formation is not affected, thus, mitofusin 2 deficiency does not deprive cells of energy supply and directly does not affect cellular viability, either. Similar stability of the ATP content was found in muscle cells depleted of *Mfn2* gene. However, in those study oxidative metabolism was substantially reduced [26,28]. Moreover, recently Son et al (2015) have shown that in MEF cells Mfn1/2 depletion facilitates the glycolytic metabolic transition through the activation of the Ras-Raf and hypoxia-inducible factor 1α (HIF1α) signaling at an early stage of reprogramming [30]. Most recently Ding et al (2015) have shown that knockdown of the *Mfn2* gene with shRNA inhibited not only oxygen consumption, but also glycolysis and cell proliferation and reduced cellular ATP content. These data probably confirm the differences between cellular response to acute and persistent deficiency of mitofusin 2 [31]. Our data shown here indicate that stable depletion of *Mfn2* gene induces adaptive processes counteracting disorganization of cell metabolism and function and preventing severe abnormalities. Such an adaptation accompanied with seriously changed pattern of protein expressed (as we also observed–not shown) in *Mfn2*-depleted cells was suggested by other authors [32]. Therefore, in experiments focused on short-term effects of mitofusin 2 deprivation silencing of *Mfn2* gene seems a better approach. In this paper however, the question concerns long-term changes in energy metabolism which allow cells to survive and proliferate despite absence of such important protein. Changes in assembling of the mitochondrial ATP-ase seem to explain both: lower level of mitochondrial energization and slightly reduced OXPHOS efficiency compensated by increased anaerobic glycolysis as proven by substantially accelerated lactate synthesis. Interestingly, similar changes in complex V structure and activity was previously shown by Blue-Native gel electrophoresis in muscle biopsies from CMT patients with pathogenic missense mutation m.9185T>C in MT-ATP6, encoding the ATP6 subunit of the mitochondrial ATP synthase [33]. Studies on fibroblasts derived from Charcot–Marie–Tooth disease type 2A sufferers reported that impaired mitochondrial fusion was accountable for a deficiency to repair stress-induced mitochondrial DNA damage, what could at least partially response for mtDNA instability [34]. Here we suppose that increased level of TFAM and PGC-1α evidence an activation of processes preventing an excessive depletion of mtDNA (below the threshold level). To conclude, comparison of two lines of MEF cells differing in the presence of the *Mfn2* gene exclusively, has revealed important differences in some parameters of energy metabolism. However, they do not affect global cellular capacity of ADP phosphorylation and cell viability.
